# Developmental Toxic Effects of Thiram on Developing Zebrafish (*Danio rerio*) Embryos

**DOI:** 10.3390/toxics10070369

**Published:** 2022-07-04

**Authors:** Bala Murali Krishna Vasamsetti, Kyongmi Chon, Juyeong Kim, Jin-A Oh, Chang-Young Yoon, Hong-Hyun Park

**Affiliations:** Toxicity and Risk Assessment Division, Department of Agro-Food Safety and Crop Protection, National Institute of Agricultural Sciences, Rural Development Administration, Wanju-gun 55365, Korea; vbmk84@gmail.com (B.M.K.V.); kjy.sara@gmail.com (J.K.); oja5074@korea.kr (J.-A.O.); evermoo2600@korea.kr (C.-Y.Y.); honghyunpark@korea.kr (H.-H.P.)

**Keywords:** developmental toxicity, teratogen, thiram, zebrafish embryos

## Abstract

Thiram, an oxidized dimer of dithiocarbamate, has fungicidal and ectoparasiticidal roles. This study aimed to determine the effects of thiram on the development of zebrafish (ZF) embryos. The developmental toxicity test was performed in accordance with the OECD 236 test guidelines, and ZF embryos were subjected to several thiram concentrations and a DMSO (0.01%) control. Subsequently, embryo mortalities and developmental anomalies were evaluated at different hours post fertilization (hpf). Thiram was highly toxic to ZF, with calculated median lethal concentrations (LC_50_) of thiram at 48 and 96 h as 13.10 ± 2.17 and 8.87 ± 2.09 μg/L, respectively. Thiram-treated embryos/larvae exhibited a variety of deformities, such as abnormal somites, reduced eye pigment, abnormal tail shape, yolk sac edema, hatching defects, and curved spines, with a median effective concentration (EC_50_) of 3.88 ± 1.23, 5.04 ± 1.82, 6.23 ± 0.92, 5.24 ± 2.22, 1.39 ± 0.25, and 2.60 ± 0.82 μg/L, respectively. Teratogenic index (TI) values ranged from 1.42 to 6.66 for the scored deformities. At 48 hpf, the average heartbeat of the control group was 177.20 ± 5.63 per minute, while the highest thiram-treated group (40 μg/L) was 99.50 ± 18.12 per minute. In addition, cardiac-related issues, such as pericardial edema and abnormal blood flow, were observed in thiram-treated ZF embryos. Overall, these findings suggest that thiram is teratogenic to ZF.

## 1. Introduction

Fungicides are indispensable for crop protection since they protect plants by killing fungi or inhibiting their growth [1]. Moreover, fungicides enter water bodies and adversely affect aquatic life, due to their regular and prophylactic use [1]. Dithiocarbamate (DTC) fungicides are non-systemic pesticides that have been used to manage numerous fungal diseases of crops and ornamentals since the 1940s. Animal and fish studies have reported DTCs as teratogenic, mutagenic, carcinogenic, and neurotoxic, and they possibly share a common toxicity mechanism [2,3,4,5,6,7,8,9].

Thiram is a DTC fungicide that primarily protects fruits, vegetables, ornamentals, and turf [2]. In addition, it is used in the rubber industry as an accelerator and vulcanization agent [10]. Thiram is one of the most widely used fungicides in agriculture, with an estimated annual average use of 140 tons (EPest-high, 1998–2018) in the US [11]. In South Korea, the amount of thiram imported in 2013–2016 was approximately 150 tons [12]. Under field conditions, thiram residual levels in water have been estimated to be 0.27–2.52 mg/L after the application of commercial thiram fungicide on oil palm nursery plots [13]. Thiram is a hazardous substance that has been associated with developmental, hepatic, and renal toxicity in animals [10]. The major toxic effects of thiram in humans are thyroid dysfunction and liver toxicity; other clinical and pathological manifestations include tachycardia, skin lesions, eye irritation, chest pain, cough, epistaxis, myocardiodystrophy, and asthenia [14,15]. In animals, thiram exhibits neurotoxic effects, such as lethargy and decreased motor activity [2]. A previous hamster study revealed that a maternal dose of 125 mg/kg thiram caused incomplete skull and spine formation, fused ribs, and heart abnormalities [16]. In addition, thiram administration in pregnant rats delayed the hardening of the cranial bones of their offspring [2], and oral administration in pregnant mice prompted higher resorptions and fetal abnormalities, such as cleft palate, micrognathia, curled ribs, and malformed bones [17]. Thiram-treated pregnant Syrian hamsters exhibited increased resorption rates and terata quantity and decreased fetal weight [4]. Dietary administration of thiram (100–500 ppm) inhibited egg-laying in chicken, quail, and partridge [18]. In accordance with the Pesticide Properties Database, thiram is toxic to *Daphnia magna* (48 h EC_50_: 0.139 mg/L) and *Oncorhynchus mykiss* (96 h LC_50_: 0.171 mg/L) [19]. Acute thiram exposure induced oxidative damage in *Daphnia magna* [20] and demonstrated toxicity in *Cyprinus carpio*, causing glycogen depletion in the liver, as well as muscle and elevated blood glucose levels [6]. Studies have linked embryonic thiram exposure to reproductive toxicity, thyroid dysfunction, spinal curvature, and abnormal craniofacial development in zebrafish (ZF) [3,21].

ZF are excellent model organisms in terms of size, economy, and ease of husbandry, and several aspects of their development are used in toxicity studies [22]. Moreover, their easy genetic modification, the possibility of omics-level analysis, and the availability of existing sequenced genomes make them ideal models for investigating toxicity mechanisms [23]. ZF produce large clutches, their embryos develop ex vivo, all major body systems form within 72 h post fertilization (hpf), and the embryos are transparent at early developmental stages; these factors result in xenobiotic exposure and simple deformity scoring [22]. ZF toxicity tests were proven to be well correlated with mammalian toxicity tests, bridging the gap between in vitro and mammalian models [24]. The fish embryo toxicity test (FET) has become increasingly popular since it allows researchers to evaluate unexpected toxicity at early developmental stages [22]. Accordingly, ZF have been successfully used to study the detrimental effects of pesticides [25], nanoparticles [26], and pharmaceuticals [27].

However, studies have not investigated the potential teratogenic effects of thiram on ZF embryos following OECD guidelines. Therefore, the current study aimed to examine dose- and time-related effects of thiram on ZF embryo/larval developmental stages by assessing post-treatment deformities, such as embryo/larval mortalities, heart rate, and body length.

## 2. Materials and Methods

### 2.1. Chemicals and Reagents

Thiram (98.4% purity) and 3,4-dichloroaniline (98% purity) were purchased from Sigma-Aldrich (St. Louis, MO, USA). Thiram stock solutions were prepared in dimethyl sulfoxide (DMSO) and stored at −20 °C for further use. Test solutions (40, 20, 10, 5, 2.5, 1.25, and 0.625 μg/L) were prepared by dissolving the required volume of stock solutions in E3 buffer (290 µg NaCl; 8.3 µg KCl; 48 µg CaCl_2_; 81.5 µg MgCl_2_ to each mL of deionized water and 0.1% methylene blue; pH 7.2). All the chemicals used in this investigation were purchased from Sigma-Aldrich (St. Louis, MO, USA) unless otherwise stated.

### 2.2. Zebrafish Maintenance and Embryo Collection

Parameters for maintaining fish stocks and collecting embryos were conducted in accordance with previously reported methods [28,29]. Briefly, the fish were housed in a glass aquarium (50 L capacity), which was supplied with dechlorinated tap water and aeration and filtering equipment. The photoperiod and temperature conditions were 14 h of light/10 h of darkness and 26 ± 1 °C, respectively. The ZF were fed dry flakes or live food (brine shrimp and blood worms) 2–3 times per day.

Mass spawning in a male-to-female ratio of 1:1 was used to harvest embryos. Similar to spawning, the same photoperiod and slightly higher temperature (27.0 °C) conditions were employed for mass spawning. The fertilized eggs were collected the next day after 30 min of light exposure. The embryos were washed thoroughly in E3 media to remove debris that had adhered to its surface.

### 2.3. Toxicity Assay

Tests were carried out in accordance with OECD guideline 236 (FET) [30]. Within 3.0 hpf, the required fertilized embryo quantity was obtained and placed in a Petri plate (100 mm) containing various thiram doses (40, 20, 10, 5, 2.5, 1.25, and 0.625 μg/L). Toxicity assays were performed in 24-well plates (SPL Life Sciences, Pocheon, Korea): Two plates for each of the seven thiram test doses, two plates for the positive control (3,4-dichloroaniline, 4 mg/L), and two plates for the DMSO (0.05%) control. The plates were inoculated with one egg per well and 2 mL of test solution in 20 wells, and 2 mL of E3 buffer in 4 wells (internal control). Half of the test solutions were replaced every 24 h for 96 h; subsequently, the plates were incubated until 144 hpf without any test solution replacement. Each experiment contained two 24-well plates per test condition, and the experiments were replicated three times (*n* = 3).

### 2.4. Deformities Scoring and Calculation

Embryo mortalities and morphological anomalies were studied using a stereomicroscope (Stemi 508, Zeiss, Oberkochen, Germany) at 24, 48, 72, and 96 hpf. Abnormal somites (AS) and abnormal tail detachment (ATD) were measured at 24 hpf; abnormal eye pigmentation (AEP), abnormal tail morphology (ATM), and tail blood flow were assessed at 48 hpf; pericardial edema (PE) and yolk sac edema (YE) were evaluated at 72 hpf; and unhatched eggs were scored at 72 and 96 hpf. Since most of the studied dosages recorded low hatching success of embryos up to 96 hpf, body length and spinal curvature (SC) were measured at 144 hpf in independent experiments, and additional embryos were used, where necessary, to obtain sufficient scorable embryos.

Based on the embryo mortality data, median lethal concentrations (LC_50_) of thiram at 48 and 96 hpf were calculated. The deformity percentage was calculated based on the total number of living embryos during the deformity scoring period. The percentage data were used to calculate median effective concentrations (EC_50_) for each deformity. The teratogenic index (TI) of each data type was calculated by dividing the LC_50_ value (96-hpf) by the EC_50_ deformity value (LC_50_/EC_50_).

### 2.5. Heart Rate Analysis

At 48 h after thiram treatment, heartbeats were assessed under a microscope. The heart beats of each embryo were counted for 20 s, and the obtained data were used to determine heartbeats per minute. Three independent experiments (*n* = 3) were performed, and each time, 10 embryos per test dose were assessed.

### 2.6. Body Length Survey

Body length (mouth tip to tail fin end) was measured using OptiView 3.7 software (Korealabtech, Seongnam, Korea) under a stereomicroscope. The body length survey was conducted three times (*n* = 3) at 144 hpf, with 10 fish per test condition.

### 2.7. Spine Deformities Scoring

The spine anomalies were assessed using a stereo microscope at 144 hpf. The experiment was carried out three times (*n* = 3) with 10 fish in each test condition.

### 2.8. Data Analysis

GraphPad Prism software version 5.0 (GraphPad Software, San Diego, CA, USA) was used to determine LC_50_ and EC_50_ values. The data are presented using mean and standard deviations (SD). An unpaired *t*-test was conducted to examine the statistical significance of the control and thiram treatments. The dose effects were evaluated using one-way ANOVA. Statistical significance was defined as *p* < 0.05.

## 3. Results

### 3.1. Acute Toxicity of Thiram

The thiram toxicity test was performed in accordance with OECD TG 232, and embryo mortality was estimated after exposure to increasing doses of thiram up to 96 hpf. The results revealed that thiram-induced mortality is dose- and time-dependent (Table 1). Although internal controls demonstrated less than 5% embryonic mortality at 96 hpf, 4-dichloroaniline treatment had approximately 60% embryonic lethality (data not shown), as required for test validity. The control and groups treated with 0.625 μg/L thiram exhibited less than 3% mortality by 96 hpf (Table 1). The cumulative mortality of the treated groups at 96 hpf are presented in Table 1. Thiram was highly toxic to ZF, with LC_50_ values of 13.10 ± 2.17 and 8.87 ± 2.09 μg/L at 48 and 96 hpf, respectively (Table 2).

### 3.2. Developmental Toxicity of Thiram

**Figure 1:**
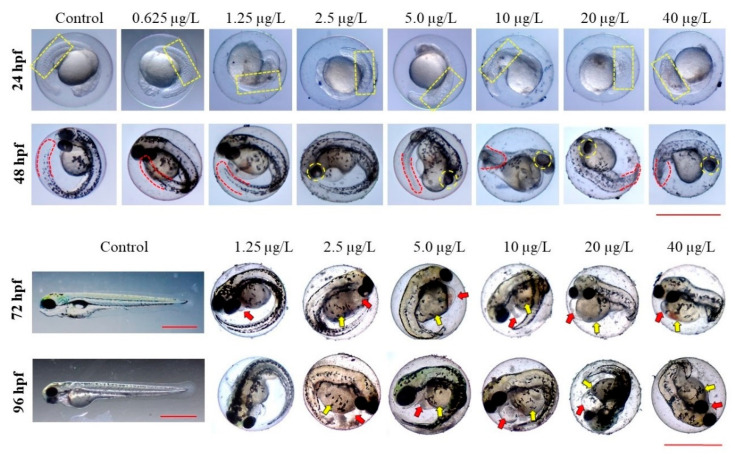
Representative images showing thiram-induced deformities at 24, 48, 72, and 96 hpf. Schemes follow the same formatting. Yellow dotted square: Somites; red dotted line: Shape of the tail; yellow dotted circle: Eyes; red arrow: Pericardial edema; yellow arrow: Yolk sac edema. Scale = 1.0 mm.

To investigate the developmental toxicity of thiram to ZF, morphological abnormalities and malformation rates were scored at 24, 48, 72, 96, and 144 hpf. During the test period, embryos/larvae treated with DMSO and 0.625 μg/L thiram showed normal growth and phenotypes (Table 1 and Figure 1). In contrast, embryos/larvae treated with 1.25 μg/L thiram and above exhibited multiple developmental deformities in a dose-dependent manner. Data collected during the morphological endpoint investigation are summarized in Table 1. Thiram induced a wide range of abnormalities in the somites, eyes, tails, hearts, yolk sacs, and spines of ZF. Some of the malformations noticed during the study are represented in Figure 1.

However, ZF treated with DMSO and 0.625 μg/L thiram showed normal somites, while those treated with 1.25 μg/L and higher thiram showed AS (*p* < 0.001) (Table 1 and Figure 1). In DMSO-treated embryos, the tail detachment was normal, while abnormal tail detachment (ATD) was random at lower doses of thiram, but exhibited a statistical significance compared with controls at the maximum test dose of 40 μg/L (*p* < 0.01), where about 30% of embryos showed a lack of tail detachment (Table 1).

AEP was observed in ZF after thiram exposure (*p* < 0.001) (Table 1 and Figure 1). Embryos treated with 1.25 μg/L thiram demonstrated reduced retina pigmentation in a dose-dependent manner at 48 hpf; when compared with controls, ZF treated with 5 μg/L thiram and above had smaller eyes. Thiram-treated embryos showed ATM (*p* < 0.001). ATM increased in a dose-dependent manner at thiram concentrations above 1.25 μg/L (Table 1). Thiram treatment caused hatching failures at 72 and 96 hpf (*p* < 0.001). The embryos of the DMSO and 0.625 μg/L thiram-treated groups hatched normally at 72 and 96 hpf (Table 1 and Figure 1). Whereas, embryo hatching was inhibited by 2.5 μg/L and above (*p* < 0.001), with less than 10% hatching at 96 hpf.

**Figure 2:**
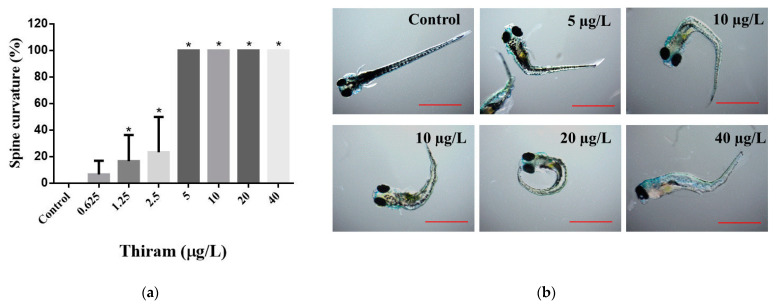
Spine curves of zebrafish induced at designated doses of thiram. (**a**) Graph shows the percentages of spine curves scored at 144 hpf. Data shown as mean ± SD were obtained from six plates (three independent experiments). Statistical differences were analyzed using an unpaired *t*-test; * *p* < 0.05. (**b**) Representative images showing thiram-induced spine curves at 144 hpf. Scale = 1.0 mm.

At 144 hpf, control groups had normal spine structure, and spine abnormalities of embryos exposed to 0.625 μg/L thiram did not differ significantly, compared with the control group (Figure 2). At 1.25 μg/L and above concentrations, the effect of thiram on spine structures differed significantly, compared with the control. In addition, at doses of 5 μg/L and above, 100% of the ZF larvae displayed spine deformities (*p* < 0.001). Thiram caused kyphosis (outward spinal curvature) and scoliosis (three-dimensional rotation or sidewise spinal curvature) in ZF. Moreover, wavy distortions of the notochord were evident in thiram-treated ZF (Figure S1).

Table 2 shows the EC_50_ and TI values for each phenotype evaluated in this study. Hatching inhibition was the most severe deformity observed with an EC_50_ of 1.38 ± 0.24 μg/L, followed by spine curvature, abnormal somites, abnormal tail blood flow, reduced eye pigment, yolk sac edema, abnormal tail morphology, pericardial edema, and no tail detachment, with EC_50_ values of 1.38 ± 0.24, 2.60 ± 0.82, 3.88 ± 1.23, 4.25 ± 1.53, 5.04 ± 1.82, 5.24 ± 5.55, 6.23 ± 0.92, 6.67 ± 5.55, and 32.50 ± 10.66 μg/L, respectively. Except for tail detachment, all the scored deformities showed EC_50_ values that were considerably lower than the 96 h LC_50_, demonstrating that thiram is a teratogen to ZF.

### 3.3. Effects of Thiram on ZF Cardiac Development and Function

**Figure 3:**
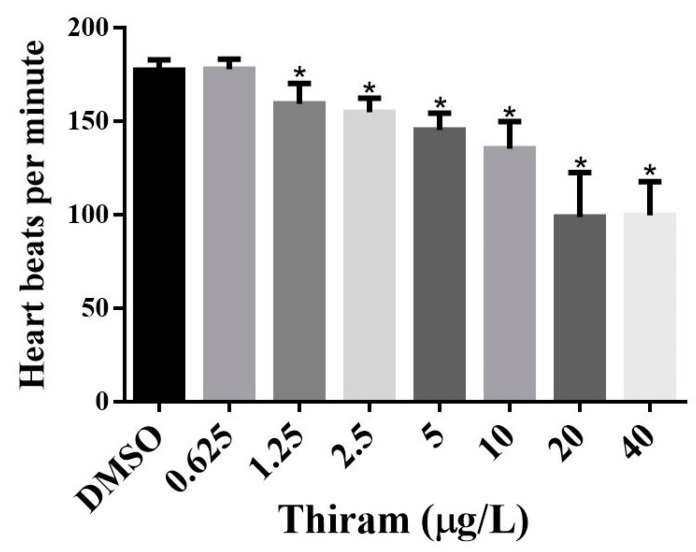
Average heartbeats per minute at designated concentrations of thiram. The heart beats were counted at 48 hpf. Data shown as mean ± SD were obtained from six plates (three independent experiments). Statistical differences were analyzed using an unpaired *t*-test; * *p* < 0.05.

To understand the effects of thiram on ZF heart development and function, pericardial edema (PE), blood flow rate, and heartbeats were scored in the control and thiram-treated groups. Thiram-treated ZF showed a significant dose-dependent increase in PE (*p* < 0.001) (Table 1 and Figure 1) at 2.5 μg/L thiram dose and gradually increased with increasing doses until it reached approximately 90% at 20 μg/L thiram dose. The thiram-treated groups exhibited abnormal tail blood flow compared with the control group (*p* < 0.001) (Table 1), with thiram treatments significantly affected even at the dose of 1.25 μg/L; approximately 8% of the embryos had consistent but reduced blood flow velocity compared with the control. At concentrations above 1.25 μg/L thiram, blood flow velocity decreased further, making it unstable and affecting approximately 90% of embryos at concentrations above 10 μg/L. Thiram had a dose-dependent effect on cardiac function (*p* < 0.001); heart rate was reduced by 10% at 0.625 μg/L thiram. Heart rate decreased further with increasing thiram doses and showed a significant reduction of approximately 44% ZF treated with 40 μg/L thiram (Figure 3). The average heart rate of the control group at 48 hpf was 177.20 ± 5.63 beats per minute (bpm), and the groups treated with 0.625, 1.25, 2.5, 5, 10, 20, and 40 μg/L thiram were 177.75 ± 5.32, 159.20 ± 0.86, 154.80 ± 7.46, 145.20 ± 9.11, 135.30 ± 14.45, 98.80 ± 23.66, and 99.50 ± 18.12 bpm, respectively.

### 3.4. Effects of Thiram on Overall ZF Growth

**Figure 4:**
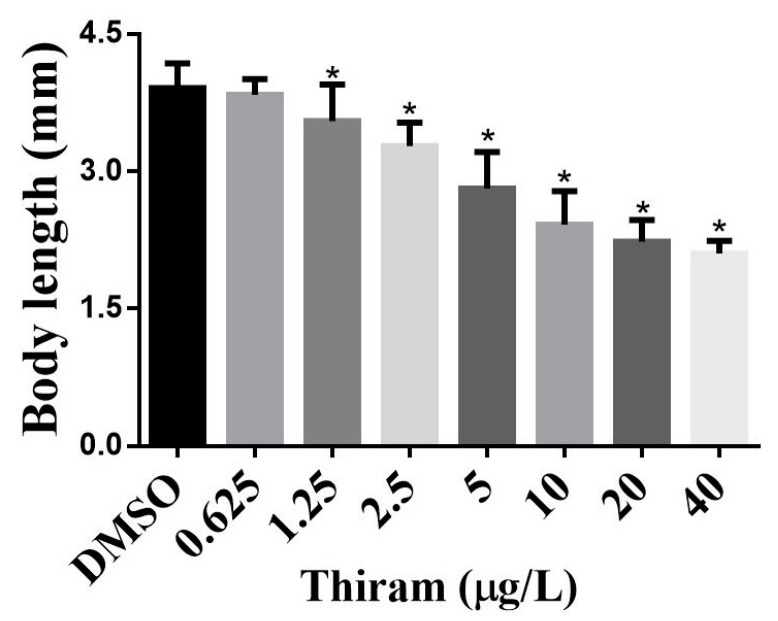
Average body length of zebrafish at designated concentrations of thiram. The body lengths were measured at 144 hpf. Data shown as mean ± SD were obtained from six plates (three independent experiments). Statistical differences were analyzed using an unpaired *t*-test; * *p* < 0.05.

Effects of thiram on overall growth were evaluated by measuring the body lengths of the controls and thiram-treated groups at 144 hpf. The average body lengths of the thiram-treated ZF are shown in Figure 4. The average body length of the control group was 3.91 ± 0.27 mm, but the average body lengths of the 0.625, 1.25, 2.5, 5, 10, 20, and 40 μg/L thiram-treated ZF were 3.84 ± 0.17, 3.55 ± 0.40, 3.28 ± 0.26, 2.81 ± 0.40, 2.42 ± 0.37, 2.23 ± 0.24, and 2.10 ± 0.14 mm, respectively. The body length of thiram-treated ZF was reduced in a dose-dependent manner (*p* < 0.001), suggesting that thiram affects the overall growth of ZF.

## 4. Discussion

In this study, the toxic effect of thiram on ZF was investigated by evaluating mortalities and developmental abnormalities. The results showed that thiram treatment caused numerous developmental defects, including abnormal somites, abnormal eye pigmentation, abnormal tail detachment, yolk sac edema, pericardial edema, hatching failure, bradycardia, spinal curvature, and notochord deformities, at concentrations not lethal to larvae (Table 1). These results are consistent with previous studies showing that DTCs, such as ziram [31], propineb [5], thiram [21], and maneb [8], induced developmental abnormalities in ZF.

Under optimal conditions, ZF embryos hatch between 48 and 72 hpf, with almost all hatching at 96 hpf [32]. Hatching failure was the most severe toxic effect of thiram on ZF, with TI values greater than 6 (Table 1). Consistent with these results, Chen et al. reported that exposure of ZF embryos to thiram at different developmental stages inhibited hatching, with exposure at an earlier developmental stage (2 hpf) as more hazardous [21]. In accordance with Chen et al., hatching failure was approximately 30% when 2.0 hpf embryos were exposed to 0.01 μM thiram for 1 h. However, the present study’s results showed that longer exposure to thiram (up to 96 hpf) at a similar dose (2.5 μg/L) and developmental stage (less than 3.0 hpf) resulted in more than 90% hatching failure (Table 1). Therefore, in addition to the stage of embryo exposure, exposure duration is possibly a critical factor that impacts hatching success. It is conceivable that hatching inhibition is one of the common toxicity mechanisms of DTCs, particularly in ZF, since other DTCs, such as maneb [8] and ziram [31], have shown hatching failure effects. In addition, at 144 hpf, at least 85% of embryos hatched at thiram doses of 2.5 μg/L or less; however, at doses greater than 2.5 μg/L, more than 90% of embryos remained in their chorions (data not shown). Since early ZF embryonic development depends entirely on limited amounts of maternally deposited egg yolk for nutrition [33], further hatching delay or prevention may be detrimental [34]. Reduced hatchability or hatching failure in response to a toxin may be justified by different toxic mechanisms, such as inhibition of enzymes necessary for the digestion of the chorion or the inability of developing larvae to break open the chorion. Hatching failures could be caused by disruption of protease secretion necessary for chorion digestion [35]. Morphological abnormalities, such as tail malformations and spine curves, may limit the embryo’s ability to break the chorion since ZF larvae with tail abnormalities showed hatching defects [23,28,29]. Disruption of mitochondrial bioenergetics is possibly responsible for hatching delays in ZF embryos treated with DTC [8]. Hatching is an important element of reproduction; therefore, interruptions seriously impact the population and natural ecosystem [36].

The heart appears to be the primary target of developmental toxicity in ZF since it is the first functioning organ to develop during embryogenesis [37]. As evidenced by its potential to induce pericardial edema, changes in blood flow, and bradycardia (Figure 1 and Table 1), thiram can be considered a cardiotoxic agent affecting both cardiac development and functioning. Thiram-treated groups exhibited edema signs in the early stages of development (24 hpf), and the size of PE further increased gradually. The incidence of PE also increased with the thiram dose, with over 90% of the embryos exhibiting PE at a dose of 20 μg/L (Figure 1 and Table 1). The results match the findings by Chen et al., who revealed that thiram exposure induces PE at a 0.01 μM dose [21]. Several DTCs have been evaluated for cardiac toxicity in ZF embryos; for instance, ziram reportedly causes PE, which is consistent with this study’s results [38]. Similarly, a high incidence of PE was reported in ZF treated with propineb, another DTC [5]. Therefore, exposure to DTCs in the early developmental stages may have deleterious effects on ZF cardiogenesis. Thiram disrupted blood flow velocities in ZF (Table 1), indicating vascular abnormalities or myocardial problems [39]. Blood flow was stable at lower doses although the flow rate was low compared with the controls, and at higher doses blood flow rates became lower and unstable, suggesting that thiram affects ZF cardiovascular development. Finally, ZF treated with thiram had decreased heart rates compared with controls (Figure 3), indicating that thiram affects cardiac function. Cao et al. suggested that failure of mitochondrial bioenergetics could be related to reduced heart rates of DTC-treated ZF [31]. Thiram reportedly affects vascular development by inhibiting blood vessel formation, disrupting angiogenesis or weakening endothelial cell adhesion, migration, proliferation, and microtubule formation [18]. Further research is required to identify the exact mechanism of thiram-induced cardiotoxicity.

Spinal curvature anomalies significantly impact aquatic life, as fish with curved spines experience swimming difficulties [23,28,29] that may interfere with feeding and predatory behaviors. Spinal abnormalities, such as scoliosis, kyphosis, and lordosis, were observed in thiram-treated ZF (Figure S1). In addition, notochord distortion was noted in the thiram-treated groups (Figure S1). These results are consistent with those of Lulla et al. (2016), Chen et al. (2018), and Cao et al. (2019), who found that DTC fungicides, namely ziram, thiram, and maneb, cause spinal curvature and notochord distortion in ZF [8,21,38]. Thiram has been shown to influence skeletal development by interfering with Zn^2+^ and Cu^2+^ ion absorption in the intestines, inhibiting liver-related enzymes involved in bone metabolism, suppressing chondrocyte differentiation, reducing osteoblast differentiation, and inhibiting hypertrophic band formation in the growth plate [18].

Notably, another adverse effect of thiram on ZF was ocular toxicity. Compared with the control groups, thiram-treated ZF accumulated less retinal pigmentation at 48 hpf, and most ZF treated with thiram at 5 μg/L and above exhibited smaller eyes (Figure 1 and Table 1). In accordance with Park et al. (2021), propineb, a DTC fungicide, causes ocular toxicity in ZFs and leads to the reduction in eye size [5]. Moreover, pesticides cause decreased retinal pigmentation [29] and alter many biological processes relevant to visual system development and perception [23].

In addition to developmental abnormalities, thiram-exposed fish displayed an overall growth reduction (Figure 1), as demonstrated by the dose-dependent decrease in body length (Figure 4). Other DTCs, such as propineb and maneb, have demonstrated similar effects on ZF development, correlating with these findings [5,8]. At the molecular level, pesticides affect a wide spectrum of gene expression and biological processes in ZF [23]. Therefore, the overall growth suppression is possibly caused by multiple perturbed molecular mechanisms occurring in response to thiram. Several mechanisms for DTC-induced deformities in ZF have been proposed. Since DTCs are copper (Cu) chelators, the documented teratogenic effects are most likely due to their ability to chelate Cu, resulting in insufficient availability of Cu to Cu-dependent proteins [7]. For instance, Cu addition reduced the teratogenic effects of DTCs, such as thiram and disulfiram, in a previous study [9]. DTCs directly inhibit Cu-dependent enzymes, for example, lysyl oxidase (LOX) inhibition was observed in thiram- and disulfiram-treated ZF [3]. A study using representative molecules from all DTC subclasses and various degradation products discovered that DTCs alter spatiotemporal gene expression of fibril-forming collagen type II α (col2α1) [9], which is an important component of the surrounding layer that supports the developing notochord. Another study observed that exposure to DTCs disrupts collagen fibril organization in the notochord sheath of developing ZF embryos [3]. Alterations in mitochondrial bioenergetics were postulated as a DTC toxicity mechanism in ZF [8]; neurogenesis-related genes, such as oligodendrocyte lineage transcription factor 2 (olig2), neurogenin 1 (neurog1), RY-box transcription factor 2 (sox2), SRY-box transcription factor 10 (sox10), and ISL LIM homeobox 1 (isl1), were downregulated in propineb-treated ZF [5], suggesting that neurogenesis disruption is another toxicity mechanism associated with DTCs in ZF. Apart from these effects, thiram has demonstrated angiogenesis inhibition, promotion of immunological damage, oxidative stress, and neurodegeneration [18]. Therefore, there are several toxicity mechanisms implicated in DTC exposure, and further studies are required to fully understand the molecular mechanism of thiram toxicity.

## 5. Conclusions

The study of thiram toxicity in aquatic environments is important due to the widespread use of thiram in agriculture. The present study revealed that early-life exposure to thiram interferes with the proper growth and development of ZF. Thiram toxicity led to various developmental abnormalities at doses below the lethal range, indicating its teratogenic potential. The toxic effects of thiram on ZF were dose- and time-dependent and were comparable to the toxic effects of other DTC fungicides. Thiram has effects on ZF at the nanomolar range, thus necessitating the investigation of thiram disposal in aquatic environments. Further research is required to better understand how thiram causes toxicity in ZF.

## Figures and Tables

**Table 1:** Deformity percentages observed at 24, 48, 72, and 96 h after thiram treatment.

	**Thiram (μg/L)**	**Mortality (%)**	**No Tail Detachment (%)**	**Abnormal Somites (%)**	**Abnormal Eye Pigmentation (%)**	**Abnormal Tail Morphology (%)**	**Abnormal Tail Blood Flow (%)**
**24 hpf**	0	0.00 ± 0.00	0.00 ± 0.00	0.83 ± 2.04			
	0.625	0.83 ± 2.04	0.00 ± 0.00	0.00 ± 0.00			
	1.25	**4.17 ± 4.92**	**5.27 ± 3.34**	**11.01 ± 13.14**			
	2.5	**5.83 ± 4.92**	0.88 ± 2.15	**43.69 ± 10.18**			
	5	**2.50 ± 2.74**	0.83 ± 2.04	**70.26 ± 29.09**			
	10	**12.50 ± 11.73**	1.28 ± 3.14	**69.87 ± 26.20**			
	20	**16.67 ± 6.06**	1.91 ± 2.96	**88.41 ± 9.98**			
	40	**19.17 ± 9.70**	**30.70 ± 15.46**	**93.52 ± 15.58**			
**48 hpf**	0	1.67 ± 2.58			0.83 ± 2.04	0.00 ± 0.00	0.00 ± 0.00
	0.625	0.83 ± 2.04			0.83 ± 2.04	0.00 ± 0.00	1.67 ± 4.08
	1.25	**8.33 ± 2.04**			**5.36 ± 4.71**	**2.68 ± 2.94**	**8.50 ± 8.88**
	2.5	**7.50 ± 5.24**			**29.28 ± 15.13**	**9.34 ± 8.85**	**33.09 ± 22.58**
	5	**7.50 ± 6.12**			**38.52 ± 16.28**	**21.16 ± 15.32**	**46.85 ± 18.56**
	10	**20.83 ± 10.21**			**87.29 ± 13.49**	**89.53 ± 4.65**	**92.03 ± 4.75**
	20	**45.00 ± 10.49**			**100.00 ± 0.00**	**98.72 ± 3.14**	**98.72 ± 3.14**
	40	**60.83 ± 9.17**			**100.00 ± 0.00**	**97.62 ± 5.83**	**97.62 ± 5.83**
	**Thiram (μg/L)**	**Mortality (%)**		**Unhatched Eggs** **(%)**	**Pericardial Edema** **(%)**	**Yolk Sac Edema** **(%)**	
**72 hpf**	0	1.67 ± 2.58		7.63 ± 10.49	3.33 ± 4.08	2.50 ± 2.74	
	0.625	0.83 ± 2.04		3.38 ± 4.11	3.38 ± 4.11	4.25 ± 5.04	
	1.25	**8.33 ± 4.08**		**43.11 ± 25.51**	14.89 ± 14.89	**8.34 ± 6.82**	
	2.5	**8.33 ± 5.16**		**91.06 ± 8.44**	**37.41 ± 29.93**	**22.04 ± 16.55**	
	5	**10.83 ± 7.36**		**95.83 ± 6.45**	**48.50 ± 31.66**	**53.44 ± 29.18**	
	10	**26.67 ± 8.76**		**95.83 ± 10.21**	**66.01 ± 20.54**	**77.85 ± 22.52**	
	20	**58.33 ± 9.83**		**100.00 ± 0.00**	**96.82 ± 4.94**	**98.48 ± 3.71**	
	40	**76.67 ± 12.11**		**100.00 ± 0.00**	**81.48 ± 29.54**	**100.00 ± 0.00**	
**96 hpf**	0	2.50 ± 2.74		0.83 ± 2.04			
	0.625	0.83 ± 2.04		0.88 ± 2.15			
	1.25	**8.33 ± 4.08**		**31.90 ± 14.17**			
	2.5	**11.67 ± 6.83**		**92.56 ± 9.53**			
	5	**23.33 ± 14.72**		**97.22 ± 6.80**			
	10	**54.17 ± 8.61**		**96.30 ± 9.07**			
	20	**81.67 ± 6.83**		**97.22 ± 6.80**			
	40	**95.00 ± 3.16**		**100.00 ± 0.00**			

Data shown as mean ± SD were obtained from six plates (three independent experiments). Statistical analysis was performed using an unpaired *t*-test. *p* < 0.05 is considered statistically significant and shown as bold text. Hpf: Hours post fertilization.

**Table 2:** Median lethal concentration (LC_50_), median effective concentration (EC_50_), and teratogenic index (TI) values obtained after thiram treatment.

Deformity	Time (hpf)	LC_50_ (μg/L)	EC_50_ (μg/L)	Teratogenic Index (96 hpf LC_50_/EC_50_)
Mortality	48	13.10 ± 2.17	-	-
Mortality	96	8.87 ± 2.09	-	-
Abnormal somites	24	-	3.88 ± 1.23	2.37 ± 0.46
Reduced eye pigment	48	-	5.04 ± 1.82	1.84 ± 0.40
Abnormal tail morphology	48	-	6.23 ± 0.92	1.42 ± 0.24
Abnormal tail blood flow	48	-	4.25 ± 1.53	2.18 ± 0.44
Unhatched embryos	72	-	1.38 ± 0.24	6.66 ± 2.22
Pericardial edema	72	-	6.67 ± 5.55	2.08 ± 1.28
Yolk sac edema	72	-	5.24 ± 5.55	1.89 ± 0.76
Unhatched embryos	96	-	1.39 ± 0.25	6.60 ± 2.10
Spine curvature	144	-	2.60 ± 0.82	3.68 ± 1.33

Data shown as mean ± SD were obtained from six plates (three independent experiments).

## Data Availability

The data presented in this study are available in the article or Supplementary Materials.

## Supplementary Materials

The following supporting information can be downloaded at: https://www.mdpi.com/article/10.3390/toxics10070369/s1. Figure S1: Representative images showing zebrafish notochords. Red arrows indicate notochord distortions. Scale = 1.0 mm.
