# Supplementary material for: Developmental Toxic Effects of Thiram on Developing Zebrafish (Danio rerio) Embryos
Source: Toxics. 2022 Jul 4;10(7):369. doi: 10.3390/toxics10070369 (PMC9317679; doi:10.3390/toxics10070369)
Supplement: Supplementary file 1 [file toxics-10-00369-s001.zip › toxics-1766712-supplementary.pdf]

toxics

MDPI

Supplementary Materials: Developmental Toxic Effects of Thiram on Developing Zebrafish (*Danio rerio*) Embryos

Bala Murali Krishna Vasamsetti, Kyongmi Chon, Juyeong Kim, Jin-A Oh, Chang-Young Yoon and Hong-Hyun Park

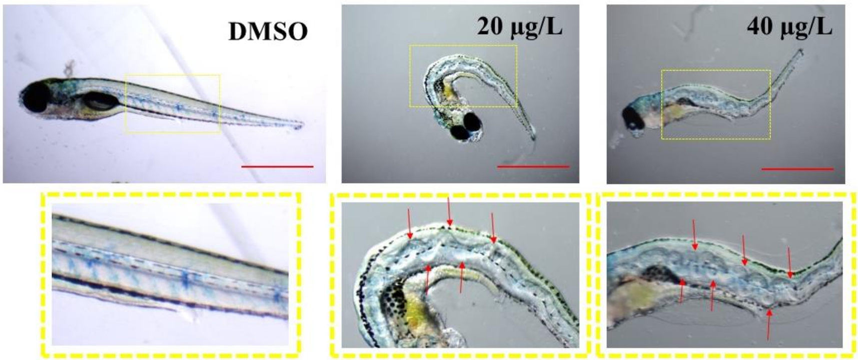

Figure S1. Representative images showing zebrafish notochords. Red arrows indicate notochord distortions. Scale = 1.0 mm.
